# Supplementary material for: Assessing the performance of methods for cell clustering from single-cell DNA sequencing data
Source: PLoS Comput Biol. 2023 Oct 12;19(10):e1010480. doi: 10.1371/journal.pcbi.1010480 (PMC10597505; doi:10.1371/journal.pcbi.1010480)
Supplement: Attachment — Submitted filename: Response_SingleCellClusteringBenchmark_v1.docx [file pcbi.1010480.s001.docx]

Dear reviewers,

Thank you so much for your time reviewing our manuscript. We greatly appreciate your time.

In the following you can find our responses for every comment the reviewers raised. The corresponding texts modified/added in the manuscript are colored in red. We mark the reviewers’ comments in italic and bold to distinguish from our responses in the following text.

***Reviewer #1: Khan and Mallory report a benchmarking study for 3 methods for clustering single—cell data. The study is well designed and should be helpful for users of such clustering methods in selecting a method for their data.***

***In the introduction the authors claim that cell clustering methods are more accurate than tree reconstruction methods but they don’t provide any references. Was this proven before?***

Sorry for the confusion. We didn’t claim that cell clustering methods are more accurate than tree reconstruction methods. Instead, we claimed that although it is desirable to have both tree reconstruction and clonal composition simultaneously, it is computationally demanding. If we perform cell clustering first, and then use the clustered cells as the subclones to reconstruct a tree, the search space of the tree will be greatly reduced. To avoid any future misunderstanding, we reword the last sentence of this paragraph as follows.

“*In all, a separate cell clustering step provides a tremendously ~~more precise and~~ smaller search space for the search of lineage tree.* ”

***When describing the simulator, the authors mention that each node was assigned an interval. What do the intervals represent?***

Answer: We maintain an interval for each node with two purposes. First, in generating the tree structure, the intervals on the nodes decide which leaf node to be split next. This was stated in this sentence in the text:

“*the chance of a leaf node being selected to split was in proportion to one minus its interval length ...* ”

The second purpose of an interval on a node is to decide the number of cells to be assigned to each leaf node.

We made these two points clearer in the text by rearranging the sentences in section “Simulate Data Intended for SNV Detection”.

***What is the beta splitting variable?***

In the process of generating a tree structure, at each round, a leaf node is selected to split (according to the interval length the leaf node has). When a leaf node is selected to split to two daughter nodes, the two daughter nodes will split this node’s interval so that the two daughter nodes’ intervals’ union will be the same as this leaf node’s interval. The Beta splitting variable determines the proportion of the left daughter nodes’ interval length over parent’s. Specifically, the left daughter node’s proportion of the parent’s node’s range is sampled from a Beta distribution, in which the two variables alpha = 0.5 and beta is the “Beta splitting variable”, the latter of which was tuned later to test the performances of the methods. Thus, Beta splitting variable controls the contrast of the sizes between the left and right daughter nodes. When the Beta splitting variable is 0.5, the left and right daughter nodes uniformly share the parent node’s interval as the mean of the sampling of the Beta distribution is 0.5 (i.e., alpha/(alpha+beta)). When the Beta splitting variable is < 0.5, the left daughter node statistically shares a bigger proportion of the parent node’s interval. For example, when the Beta splitting variable is 0.1, the mean of the sampling for the left daughter node’s interval’s proportion over the parent node’s interval is 0.5 / (0.5 + 0.1) = 0.83. Thus, the left daughter node has a higher chance to share a bigger proportion of the interval than the right daughter node. Since we distribute the cells to the leaf nodes according to each node’s interval length, Beta splitting variable’s being small would further lead to both large and small clusters coexisting in the subclone pool, and thus a larger variance of cluster sizes. To avoid future readers’ confusion on the Beta splitting variable, we rewrote this part in section “Simulate Data Intended for SNV Detection” under “Description of Simulation”, together with the explanation of the “interval” since these two are closely related with each other.

***Were the SNV assigned randomly?***

The SNVs are assigned to the edges of the tree such that the number of SNVs on each edge is proportional to the simulated edge length. For example, if a tree has three edges, e1, e2 and e3, whose lengths are 0.2, 0.3 and 0.5, respectively. Suppose we have 100 SNVs to be assigned. We assign 20 SNVs to e1, 30 to e2 and 50 to e3. In our simulation, SNVs do not have the actual biological meaning, therefore we do not care about the order of SNVs while assigning them to the edges in generating the simulated data. These SNVs’ presence and absence will be shown in the genotype G matrix. The accuracy of the recovered SNV’s genotype in each cell from the five methods was examined in our performance evaluation.

To make the whole process of assigning SNVs more clearly in the text, we added the following text to our manuscript in section “Simulate Data Intended for SNV Detection” under “Description of Simulation”:

“*Once the splitting is finished and the tree structure has been formed, for each edge, we sample its edge length from an exponential distribution whose lambda is 1. Afterwards we assign a given number of cells to the leaf nodes in proportion to each leaf node's interval length, and a given number of SNVs to all edges on the tree in proportion to each edge's length*.”

***The authors need to describe the computing resources that were used for the reader to understand the described runtimes.***

Our jobs run alternatively on either of these two types of processors: Intel(R) Xeon(R) CPU E5-2680 V4 @ 2.40GHz and Intel(R) Xeon(R) Gold 6248 CPU @ 2.50GHz. We added this to our manuscript in the section “Usage of SCG, SCClone, BnpC, RobustClone, SCITE and SBMClone”.

***Was Cross—validation performed when calculating performance metrics? This should be done and also clarified.***

Cross-validation was not performed because there is no such a step as training a model in all six methods. None of the computational tools that we benchmarked learns from labeled data, mainly because most studies do not have labeled data. Thus cross-validation is not applicable in our benchmarking.

***A tabular overview of all performance metrics and all simulations would be helpful.***

We added Tables 2-9 as an overview of all performance metrics on all simulations for all the methods. If necessary, we are happy to move them to a supplemental document.

***The differences of the underlying algorithms for each method should be discussed. Do those differences explain the difference in performance?***

Thank you for pointing this out. Yes, the goal of this study is also to understand the relationship between the design of the methods and their performances. In addition to a brief introduction to each method in the “Background” section, we explained from the algorithm design’s perspective why a certain method behaves in a certain way in “Discussion”. For example, to explain the observation that RobustClone is extremely fast, we added the following text:

*“RobustClone's running time was consistently the least among all methods probably due to that 1) it does not model the latent variables such as FP and FN rates; 2) it separately recovers the genotype matrix and clusters the cells instead of doing both jointly.”*

We added explanations from algorithm design’s perspective not only for good performances, but also for bad performances. For example, we observed that SCClone is sensitive to false positive rate. We therefore added the following sentence:

*“This is probably because SCClone has a fixed initial FP rate. When the true FP rate was greater than the fixed initial FP rate (0.01), SCClone failed to correct those false positive entries.”*

***Reviewer #2: This paper assesses three methods for cell clustering and genotyping of SNVs measured in single-cell DNA sequencing data. To do so, the authors adapted their previous simulator to better control clone sizes. I have a couple of concerns that reduced my enthusiasm for this paper.***

***0. Selection of methods***

***It is unclear why tree based methods are excluded. bnpc for example includes a comparison to SiCloneFit.***

Answer: Thanks for pointing this out. We incorporated a state-of-the-art tree-based method SCITE in our benchmark experiments. This experiment helped us to understand more about SCITE’s performance on clustering the cells.

***Also, there is a recent method called SBMClone [1] that was not included in the comparison.***

Answer: Thank you for letting us know about this method. We incorporated SBMClone in our study. However, since SBMClone was designed for sparse data sets which have very low coverage, the other five methods were not designed for such data sets. We therefore simulated an ultra-low coverage large data set to evaluate SBMClone. We tried running all other five methods on this data set, but none of them gave us meaningful results within the time limit (we set the time limit to be 4 hours to have a first screen then increased it to 48 hours for the second screen). We therefore reported only SBMClone’s results on this data set. More details can be seen in sections “Simulating Ultra-low Coverage Data” under “Description of Simulation”, “Simulation Experiments for Ultra-low Coverage Data” under “The Design of Simulation Experiments”, and “Simulation Results for Ultra-low Coverage Data” under “Benchmark of Simulation Experiments”.

***Finally, the authors write:***

***"Here we skip discussing RobustClone because it does not have a friendly user interface and is not as popular."***

***I suggest rephrasing this ("not as popular").***

Answer: Thanks for the suggestion. Since the editor pointed out “*for a benchmarking paper it is important to broadly include all types of methods rather than stating that some are known to perform worse - the point of the benchmarking is to quantitatively evaluate that*”, we included RobustClone in our benchmark study in this revision.

***1. Writing needs to be improved.***

***There are several grammatical errors throughout.***

Thank you for pointing this out. We carefully read the revised manuscript and corrected as many grammatical errors as we could catch, all shown in red.

***2. Sensitivity and specificity are undefined***

***It is unclear how these two metrics are defined. Is it in terms of pairs of cells? Please elaborate. Also, please elaborate why these two metrics render genotype accuracy redundant?***

Answer: Thank you for pointing this out. We added the following sentence in the “Comparison Metrics” section.

*In particular, sensitivity measures the percentage of correctly reported 1's by the methods over the total number of 1's in the ground truth G matrix, and specificity measures the percentage of correctly reported 0's by the methods over the total number of 0's in the ground truth G matrix.*

As for why these two metrics render genotype accuracy redundant, we added the definition of accuracy in the following sentence in the “Comparison Metrics” section.

*“We also measured the genotyping accuracy which is the percentage of correctly reported 1's and 0's by the methods over the total number of entries in the ground truth G matrix … ”*

As it can be seen, accuracy is a metric combining sensitivity and specificity. Thus we thought it was enough to measure sensitivity and specificity separately without the accuracy.

***3. Doublets***

***Papers states:***

***"Thus all three methods are not doublet-aware and the measurements do not include doublet cells throughout the entire benchmark study. However, the accuracy of clustering non- doublet cells in the presence of different levels of doublet cells is still of interest and we included this study in one of our simulation experiment."***

***It is unclear whether doublets were excluded from the input, or only excluded when computing V-measure? This needs to be clarified.***

Answer: The latter is true. Doublet cells are still in the input to the methods. But when we compute V-measure, the sensitivity and specificity, we do not involve the doublet cells. We rewrote this sentence as follows to clarify this point at the end of the section “Usage of SCG, BnpC and SCClone”:

“*Thus all five methods are not doublet-aware, and the measurements do not include doublet cells throughout the entire benchmark study. However, the accuracy of clustering non-doublet cells in the presence of different levels of doublet cells is still of interest and we included this study in one of our simulation experiments, in which the input to the five methods involved doublets, whereas the evaluation metrics (V-measure, sensitivity and specificity) did not involve doublets.* ”

Further in section “Simulation Results for the Data Intended for SNV Detection” under “Benchmark of Simulation Experiments”, we added this paragraph while analyzing the results with varying doublet rate.

“*In the experiment of the varying doublet rate, the input to the five methods contained doublets to different percentages. However, since we used the singlet model for SCG, and none of the rest of the four methods explicitly report doublets, we only measured V-measure, sensitivity and specificity on non-doublet cells and excluded the doublets from these measurements. Thus this experiment examines the clustering accuracy of the non-doublet cells in the face of varying doublet rate in the data.”*

***4. Effect of coverage not assessed***

***My biggest concern is that the starting point of the simulations is a binary matrix indicating presence/absence of mutations in individual cells. This is unrealistic, as in practice one observes variant and reference read counts.***

Thank you for pointing this out. We agree that in reality, the input, instead of a binary matrix, is the variant/reference read counts at each SNV site for each cell. In addition, as the reviewer kindly pointed out, the coverage of the genome is an important variable that affects the performance of the methods. Thus the coverage and sequencing errors will affect the variant/reference read counts, which further affect the observed D matrix. We seriously considered simulating variant/reference read counts to determine the D matrix, but decided not to do so given the following three reasons.

1. All five methods that we benchmarked on the data set intended for SNV detection take the D matrix instead of the variant/reference read count as the input.
2. We want to gain a good control of the false positive rate, false negative rate and missing rate. Simulating D matrix directly from G matrix can render us the desired false positive rate, false negative rate and missing rate. However, it is indirect to control these error rates via tuning the variant/reference read count.
3. Taking into account the variant/reference read counts requires us to consider the accuracy of SNV caller that can turn the variant/reference read counts to the noisy D matrix. Some SNV callers, such as Monovar, are more sensitive in calling SNVs in scDNAseq, whereas others, such as GATK Haplotype caller, are less sensitive in calling SNVs in scDNAseq. The selection and discussion of the SNV callers is a different, although related subject from this study, and thus is out of scope of this study.

We added the following paragraphs to the text right after where we described how we simulated the D matrix in section “Simulate Data Intended for SNV Detection” under “Description of Simulation” to state this alternative way in simulation and why we chose not to do it in this way.

*“Since all five methods being evaluated on the data set simulated in the above-mentioned way (SCG, SCClone, BnpC, RobustClone and SCITE) take the noisy cell locus matrix as the input, the resulting simulated matrix (D matrix) was the input to these tools. Notice that an alternative way to simulate the input to these methods is to simulate the read counts supporting the variant and reference alleles, respectively, for each SNV, and then convert the read count to the D matrix according to the number of variant-supporting reads and total number of reads. This way of simulation is more realistic because it takes into account the coverage of the data and sequencing errors. Although this way mimics the real sequencing process better, we decided not to implement the simulator in this way because the former way that simulates D matrix from G matrix directly can give us a much better control of the false positive rate, false negative rate and missing rate. Thus we can produce a D matrix that has exactly the desired false positive rate, false negative rate and missing rate. On the other hand, turning the read count to a D matrix is nontrivial because it depends on the SNV calling algorithms. Some SNV calling algorithms, such as Monovar, utilize the read count from a group of cells to detect SNVs, and thus are sensitive in calling the SNVs even if some cells have a small number of variant-supporting reads, whereas other SNV calling algorithms, such as GATK HaplotypeCaller, are less sensitive at the SNVs that have low variant-supporting reads. However, the discussion of the upstream SNV calling algorithm is out of the scope of this study. Since all the five methods being evaluated take the D matrix instead of the read count as the input, to gain a better control of the error rates and not to dive deep into the differences of the SNV calling algorithms, we did not use the read count to determine the D matrix but directly generated the D matrix from the G matrix.”*

However, in our ultra-low coverage large scDNAseq experiment, since the coverage determines how sparse the D matrix would become and it is our varying variable, we designed our simulator that generated variant/reference read count. In this study, since the missing rate was as high as >99%, we no longer investigated false positive rate and false negative rate. Also, the way of determining whether a SNV can be observed or not was greatly simplified as in most cases a SNV was missing except when there was at least 1 variant-supporting read, in which case the corresponding entry of the D matrix was 1.

***Moreover, with more recent technologies the coverage is low (0.01-0.05X). I recommend the authors to also assess ultra-low coverage data.***

Thank you for this suggestion. We designed the simulator that can simulate the ultra-low coverage data set and varied the coverage between 0.01x and 0.05x, as seen in “Simulating Ultra-low Coverage Data” under “Description of Simulation”, “Simulation Experiments for Ultra-low Coverage Data” under “The Design of Simulation Experiments”, as well as “Simulation Results for Ultra-low Coverage Data” under “Benchmark of Simulation Experiments”. This data set mainly benchmarked SBMClone which was designed for sparse data sets. As stated above in response to the reviewer’s suggestion of adding SBMClone to our experiment, we tried running all other five methods on this data set, but none of them gave us meaningful results within the time limit. We therefore reported only SBMClone’s results on this data set.

*[1] Myers, Matthew A, Simone Zaccaria, and Benjamin J Raphael. “Identifying Tumor Clones in Sparse Single-Cell Mutation Data.” Bioinformatics 36, no. Supplement_1 (July 1, 2020): i186–93.* [*https://doi.org/10.1093/bioinformatics/btaa449*](https://doi.org/10.1093/bioinformatics/btaa449)*.*

Zahn, Hans, et al. "Scalable whole-genome single-cell library preparation without preamplification." *Nature methods* 14.2 (2017): 167-173.

Zafar, Hamim, et al. "Computational approaches for inferring tumor evolution from single-cell genomic data." *Current Opinion in Systems Biology* 7 (2018): 16-25.

Mallory, Xian F., et al. "Methods for copy number aberration detection from single-cell DNA-sequencing data." *Genome biology* 21.1 (2020): 1-22.

Gao, Ruli, et al. "Punctuated copy number evolution and clonal stasis in triple-negative breast cancer." Nature genetics 48.10 (2016): 1119-1130.

Thanks again for your constructive comments. We really appreciate them.

Best,

Xian Mallory on behalf of the authors

Feb. 16^th^, 2023
