# Supplementary material for: Assessing the performance of methods for cell clustering from single-cell DNA sequencing data
Source: PLoS Comput Biol. 2023 Oct 12;19(10):e1010480. doi: 10.1371/journal.pcbi.1010480 (PMC10597505; doi:10.1371/journal.pcbi.1010480)
Supplement: Attachment — Submitted filename: Response_SingleCellClusteringBenchmark_v2.docx [file pcbi.1010480.s002.docx]

Dear external editor and reviewer,

Thank you so much for your time reviewing our manuscript. We greatly appreciate your time.

In the following you can find our responses for the comment that the external editor raised. We mark our responses in red to distinguish from the external editor’s comments. The corresponding texts modified/added in the manuscript are colored in red. We also included a clean version of the manuscript.

**External Editor Review Comments:**

I've carefully reviewed the revised manuscript and reviews. I believe that I see the main issue and how it can be resolved. The authors respond to all of reviewer 1's suggestions. The authors respond fully including additional analyses as well as relevant changes to the text to all of reviewer 2's suggestions/critiques except "4. Effect of coverage not assessed" and "2. Sensitivity and specificity are underlined". For these two suggestions/critiques, the authors make clarifications in the text but rebut major aspects of the suggestions/critiques. The most significant of these is "4. Effect of coverage not assessed" where reviewer 2 starts this critique with "My biggest concern...". I will address this first, followed by 2. which is minor. I'll end with my brief thoughts on the manuscript as it is.

Regarding reviewer 2's point 4, reviewer 2 has a point that what a user would observe/have in hand is variant and reference read counts. The reviewer suggests simulating that as opposed to what the authors simulate and is a next step in the analysis of this kind of data which is generating a D matrix containing inferred genotypes from the read count data. The authors argue that the D matrix is what's input to each of the tools they're assessing, so they stand by simulating the D matrix and not read counts. I see both points. Here's my view of this standoff. As long as the D matrices that the authors are simulating are representative of the D matrices that would be generated by actual scDNA-seq data, then I believe the arguments the authors make are valid and their results are useful to the scDNA-seq community. They would have to redo the whole analysis from a new starting point if they had to simulate the variant/reference read counts. However, the authors don't go into great detail regarding the parameters that they used to simulate their D matrices and evaluate all of these tools. These parameters are shown in Table 9 (and used throughout the text) and the values they take are justified in one sentence in the methods (on p. 29 of the current text): "We selected the ranges for each variable mainly according to the discussion in [19]". These parameters include false positive and false negative rate which are not immediately observed by a user which supports, in part, reviewer 2's issue with this approach. The authors go into great detail about their tree-based synthetic data generation method which also appears to follow a previous publication. As long as this tree-based method and the parameter settings are representative of actual D matrices generated from actual scDNA-seq data then the authors benchmarking and conclusions should be on reasonably solid ground and useful to the scDNA-seq community. For example, they have two clear winning methods/tools, BnpC and SCG, across all parameters that they explored. So the conclusions are relatively simple and don't depend on read coverage or any of the simulated parameters (except cell number which is directly observable and affects runtime of these top two tools). If not, then reviewer 2's concerns are valid. One way to address this in a more conclusive manner, than just relying on reference [19], is to demonstrate that the simulated D matrices are representative of/effectively indistinguishable from D matrices derived from actual scDNA-seq data. This should at least be true for parameters within the range of those shown in Table 9. This could be done by randomly sampling these matrices and generating heatmaps or taking another approach. This would resolve the issue in my view.

Thank you for pointing this out. We acknowledge that our previous justification of the selection of the range of parameters was not as sound as it should have been because it included only one reference. We included a lot more references in the subsection “**Simulation Experiments for the Data Intended for SNV Detection**” under the “**Design of Simulation Experiments**” in **Methods**. With these references, we are justifying the selection of the range of false positive rate and false negative rate as these two are not immediately observed by a user. In addition, according to the exterior editor’s suggestion, we randomly sampled the matrices D, one from each parameter setting for all false positive rates and all false negative rates, and plotted the heatmaps of sampled matrices shown in Fig. 13 (varying false positive rates) and Fig.14 (varying false negative rates). We added these heatmaps to show that our tree-based simulation method and the parameter settings are representative of the D matrices generated from the actual scDNA-seq data, to be compared with the heatmaps of two human colorectal cancer patients’ single-cell sequencing datasets (Fig. 3C and Fig. 3D of [1]).

[1] Leung ML, Davis A, Gao R, Casasent A, Wang Y, Sei E, Vilar E, Maru D, Kopetz S, Navin NE. Single-cell DNA sequencing reveals a late-dissemination model in metastatic colorectal cancer. Genome research. 2017 Aug 1;27(8):1287-99.

Regarding reviewer 2's point 2, the authors argue that they effectively assess accuracy which depends on sensitivity and specificity so they show sensitivity and specificity separately without also explicitly calculating accuracy. The authors are correct. Formally, Accuracy = Sensitivity * Prevalence + Specificity * (1 - Prevalence) where Prevalence = Positives/(Positives + Negatives). By showing sensitivity and specificity separately, they show the weakest of the two which allows a bound of the Accuracy without knowing the Prevalence. The two top methods, BnpC and SCG, tend to have high values of both across parameters assessed, so this is not an issue.

If the authors demonstrate more convincingly, that the D matrices that they generate are representative of actual D matrices derived from actual scDNA-seq data, then I think they were responsive to reviews and that this paper would be useful to the scDNA-seq community.

Reviewer's Responses to Questions

**Comments to the Authors:
Please note here if the review is uploaded as an attachment.**

Reviewer #1: All my concerns have been addressed

**Have the authors made all data and (if applicable) computational code underlying the findings in their manuscript fully available?**
The [PLOS Data policy](https://urldefense.com/v3/__https:/journals.plos.org/ploscompbiol/s/materials-and-software-sharing__;!!PhOWcWs!zJxr_MyXQhRANHNeWCmLssvVXzBWrIPpPDs2R7gPbo2kFp1RbGuf-NW7wWv-EhlrA8gyZ1rjTFNFLwM$) requires authors to make all data and code underlying the findings described in their manuscript fully available without restriction, with rare exception (please refer to the Data Availability Statement in the manuscript PDF file). The data and code should be provided as part of the manuscript or its supporting information, or deposited to a public repository. For example, in addition to summary statistics, the data points behind means, medians and variance measures should be available. If there are restrictions on publicly sharing data or code —e.g. participant privacy or use of data from a third party—those must be specified.

Reviewer #1: None

PLOS authors have the option to publish the peer review history of their article ([what does this mean?](https://urldefense.com/v3/__https:/journals.plos.org/ploscompbiol/s/editorial-and-peer-review-process*loc-peer-review-history__;Iw!!PhOWcWs!zJxr_MyXQhRANHNeWCmLssvVXzBWrIPpPDs2R7gPbo2kFp1RbGuf-NW7wWv-EhlrA8gyZ1rj5U378qo$)). If published, this will include your full peer review and any attached files.


If you choose “no”, your identity will remain anonymous but your review may still be made public.


**Do you want your identity to be public for this peer review?** For information about this choice, including consent withdrawal, please see our [Privacy Policy](https://urldefense.com/v3/__https:/www.plos.org/privacy-policy__;!!PhOWcWs!zJxr_MyXQhRANHNeWCmLssvVXzBWrIPpPDs2R7gPbo2kFp1RbGuf-NW7wWv-EhlrA8gyZ1rji06pX8s$).

Reviewer #1: No
